# The effect of job strain and worksite social support on reported adverse reactions of COVID‐19 vaccine: A prospective study of employees in Japan

**DOI:** 10.1002/1348-9585.12356

**Published:** 2022-10-21

**Authors:** Natsu Sasaki, Reiko Kuroda, Kanami Tsuno, Kotaro Imamura, Norito Kawakami

**Affiliations:** ^1^ Department of Mental Health, Graduate School of Medicine The University of Tokyo Tokyo Japan; ^2^ Division for Environment, Health, and Safety The University of Tokyo Tokyo Japan; ^3^ School of Health Innovation Kanagawa University of Human Services Kawasaki Kanagawa Japan

**Keywords:** job stressors, mental health, occupational health, reactogenicity, side reactions

## Abstract

**Objectives:**

This prospective study aimed to examine the association of psychosocial working conditions with adverse reactions after receiving COVID‐19 vaccination in a sample of employees in Japan.

**Methods:**

The data were retrieved from an online panel of full‐time employees (E‐COCO‐J). The analysis included participants who were employed and were not vaccinated at baseline (June 2021) but received vaccination at a 4‐month follow‐up (October 2021). An 11‐item scale measured the adverse reactions. Four types of psychosocial working conditions (i.e., job demands, job control, and supervisor and coworker support) were measured using the Brief Job Stress Questionnaire. Multiple linear regression analyses were conducted to examine the relationship between the psychosocial working conditions and adverse reactions of COVID‐19 vaccines, adjusting for socioeconomic variables, chronic disease, the number of vaccination, type of vaccine, anxiety for adverse reactions, fear and worry about COVID‐19, and psychological distress at baseline.

**Results:**

Overall, 747 employees were included in the analysis. The average number of adverse reactions was 3.8 (standard deviation = 2.2): Arm pain (81.1%), fatigues (64.1%), muscle pains (63.3%), and fever (37.5°C+) (53.5%) were reported more frequently. Coworker support score was significantly and negatively associated with the numbers of adverse reactions (standardized *β* = −0.100, *P* = .023). Women, young age, second‐time vaccination, Moderna, and high psychological distress were significantly associated with adverse reactions.

**Conclusions:**

Employees with low coworker support may be more likely to have adverse reactions after vaccinations. The findings of this study could support that increasing workplace support may reduce adverse reactions.

## 1 INTRODUCTION

Solicited local and systemic adverse events, that is, adverse reactions after the injection of COVID‐19 vaccines, have been frequently reported.^1^, ^2^ They affect the daily life activities of the recipients. They may also be a major reason for vaccine hesitancy.^3^ The immediate and non‐specific innate immune response can produce various adverse reactions.^4^ Women, young people, second dose, heterologous prime‐boost, and individuals with previous SARS‐CoV‐2 infection are more likely to experience adverse reactions of the COVID‐19 vaccine.^5^, ^6^ Differences in adverse reactions have been attributed to increased immunogenicity to the COVID‐19 vaccine among these groups.^5^, ^7^, ^8^

Psychological factors can affect the immune system's response to the vaccine, thus the side effect.^4^ After influenza virus vaccination, for example, chronic depression was associated with excessive and prolonged inflammatory responses.^7^ Exposures to a brief stressor before receiving the typhoid vaccine amplified the inflammatory response to the vaccine.^8^ Psychological factors may also trigger short‐term adverse reactions of COVID‐19 vaccination. However, to date, no study has examined this association.

Poor psychosocial working conditions, such as high job demands, low job control,^9^, ^10^ and lack of workplace support,^11^ have been associated with immune system dysregulation.^12^, ^13^, ^14^, ^15^ Poor psychosocial working conditions often increase inflammatory markers while reducing cellular immune functions (such as NK cell activity, NK and T cell subsets, CD4^+^/CD8^+^ ratio).^16^ Thus, people working under poor psychosocial conditions may experience more adverse reactions after the COVID‐19 vaccine because of decreased innate immune response to vaccination caused by such stressful conditions.

This prospective study aimed to examine the association of job demands, job control, and lack of workplace support with adverse reactions after receiving COVID‐19 vaccination in a sample of full‐time employees in Japan.

## 2 METHODS

### 2.1 Study design and participants

**FIGURE 1:**
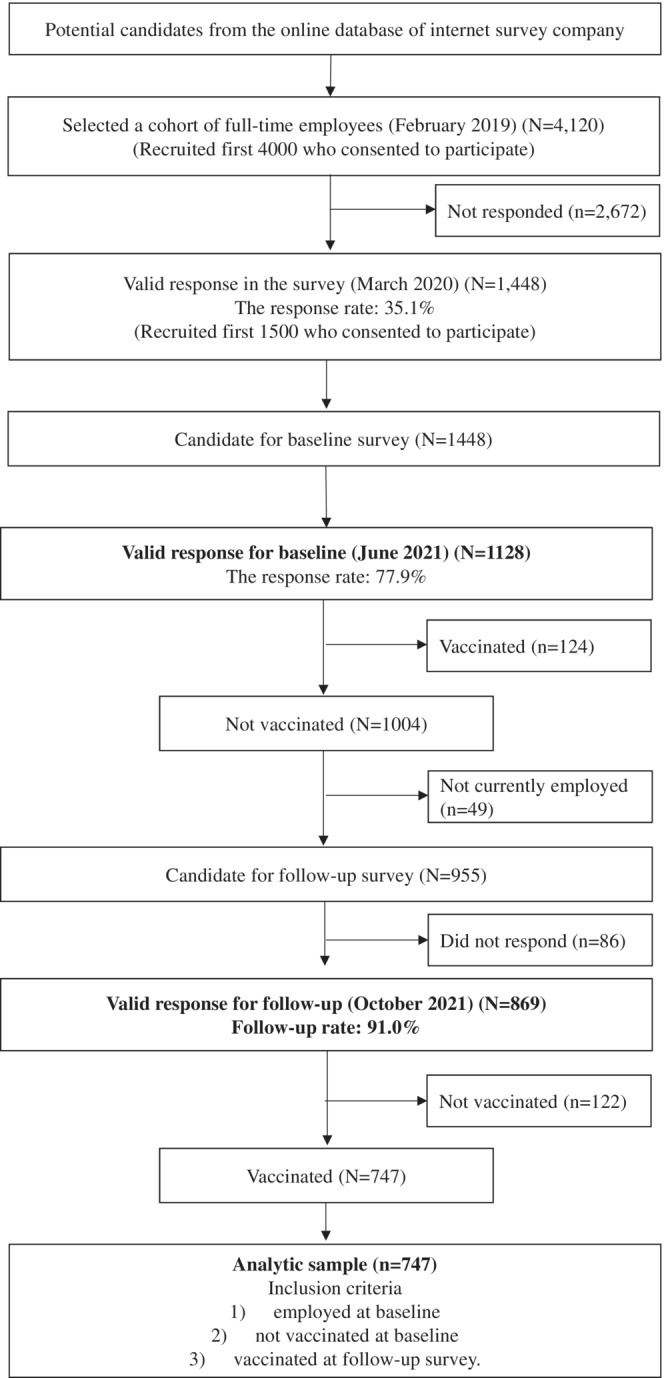
Flowchart of the participant recruitment.

The data were collected as a part of a large‐scale prospective panel study, the Employee Cohort Study in the Covid‐19 pandemic in Japan (E‐COCO‐J),^17^, ^18^ targeting a sample of full‐time employees recruited from the panel of the Japanese internet company in March 2020 (*N* = 1448). After completing six surveys (including the first survey) between March 2020 and March 2021, the seventh and eighth surveys were administered in June and October 2021, respectively. In this prospective study, the baseline variables (such as job strain and workplace support) were measured in the seventh survey (hereafter called the baseline), and the outcome variables (i.e., the side effect of the COVID‐19 vaccine) were measured in the eighth survey (hereafter called as the follow‐up). The participants' eligibility criteria were: (1) being employed at baseline, (2) not being vaccinated at baseline, and (3) being vaccinated at follow‐up. The details of the recruitment process are shown in Figure 1.

The Research Ethics Committee of the Graduate School of Medicine/Faculty of Medicine, The University of Tokyo, approved this study, No. 10856‐(2)(3)(4)(5). This study followed the Strengthening the Reporting of Observational Studies in Epidemiology (STROBE) statement: guidelines.^19^

### 2.2 Measurement variables

#### 2.2.1 Adverse reactions of COVID‐19 vaccine

A list of 11 adverse reactions of the COVID‐19 vaccine was created by referring to the report of the possible common adverse reactions reported by the Centers for Disease Control and Prevention (CDC),^20^ including arm pain/redness/swelling, fatigues/tiredness, headache, muscle pain/joint pain, chills, fevers (37.5°C+), nausea/vomit, diarrhea, lymph node pain, severe reactions to being needed medical care (e.g., anaphylaxes), and delayed local arm reactions after 7 days of vaccinations (i.e., COVID arm). We developed an 11‐item scale of adverse reactions of COVID‐19 vaccines. Participants were asked whether they had each of the listed adverse reactions within a few days after the vaccination: “Did you experience any of the following adverse reactions within 1–3 days after getting a COVID‐19 vaccine?” If respondents received only the first dose, they were asked to report their experience at that time; if they received the vaccination twice, they were asked to report their experience when their symptoms were most severe. The response options were Yes or No. The total number of adverse reactions, ranging from 0 to 11, was used as a primary outcome.

#### 2.2.2 Psychosocial working conditions

Psychosocial working conditions were assessed based on the job‐demand‐control (JDC) model and the job‐demand‐control‐support (JDCS) model, which explain the occurrence of mental strain in a workplace context.^9^, ^21^ Four components of the JDC/JDCS model, job demand (quantitative job overload), job control, coworker support, and supervisor support, were measured using the corresponding subscales of the Brief Job Stress Questionnaire (BJSQ).^22^ Each scale comprised three items, with each being rated on a 4‐point Likert‐type scale (ranging from “very much so” = 1 to “not at all” = 4 for job demands and job control and from “Extremely” = 4 to “not at all” = 1) for supervisor and coworker support. Total scores for each subscale ranged from 3 to 12, with a higher score indicating the higher degree of the corresponding component. The four components of the BJSQ showed good reliability and validity.

#### 2.2.3 Covariates

##### Anxiety about adverse reactions of vaccines

One original item was used to assess anxiety about the adverse reactions of vaccines. This item stated, “I am concerned about the effectiveness and adverse reactions of COVID‐19 vaccines,” Responses were scored on a 4‐point Likert‐type scale (ranging from 1 “Not at all” to 4 “Feel strong”). The reliability and validity were not examined.

##### Fear and worry about COVID‐19

One original item was used to assess participants' fear of COVID‐19. The item asked, “Do you feel anxious about COVID‐19?” Responses were scored on a 6‐point Likert‐type scale (ranging from 1 “Not at all” to 6 “Feel strongly”). The reliability and validity were not examined, but several papers using the same scale were published.^17^, ^23^, ^24^

##### Psychological distress

Psychological distress was measured using K6 (Kessler 6).^25^, ^26^ Respondents were asked to report how frequently in the past 4 weeks they felt nervous, hopeless, restless or fidgety, worthless, depressed, and that everything was an effort. The response options were “none of the time” = 0, “a little of the time” = 1, “some of the time” = 2, “most of the ime” = 3, or “all of the time” = 4. The total score ranges from 0 to 24. The Japanese version of K6 showed good reliability and validity.^27^ K6 performs just as well as the Composite International Diagnostic Interview (CIDI) Short Form in identifying individuals with clinically significant mental disorders.^26^

##### Demographic variables

We measured gender, age, educational attainment, marital status, occupation, chronic disease at baseline, the number of vaccination (first or second time), and type of vaccine (Pfizer/BioNTech, Moderna, or unknown) at follow‐up. Chronic disease was defined as having any physical and psychological diseases which were currently treated in the medical settings, including hypertension, diabetes, heart disease (e.g., angina, heart failure), cerebrovascular disease (e.g., cerebral infarction, cerebral hemorrhage), cancer/malignant neoplasm, respiratory disease, liver disease, kidney disease, and depression/anxiety/unstable moods. Hospital admissions or home treatment over 1 week regardless of COVID‐19 in the past 6 months was measured at the follow‐up.

### 2.3 Statistical analysis

The descriptive statistics were used to describe sociodemographic characteristics, psychosocial working conditions, and the prevalence of reported adverse reactions. We conducted multiple linear regression analyses to examine the relationship between four psychosocial factors at work (job demand, job control, coworker's support, and supervisor support) and the adverse reactions of COVID‐19 vaccines in the crude model (Model 1). The relationship was also assessed when adjusting for gender, age, educational attainment, marital status, occupation, chronic disease, the number of vaccination, and type of vaccine (Model 2) and for anxiety about adverse reactions of vaccines, fear and worry about COVID‐19 and psychological distress at baseline (Model 3). The same subgroup analysis was conducted among participants who received second vaccination.

The sample may have included participants who tested positive for COVID‐19 before the baseline or during the follow‐up, which may have confounded the results. We were not allowed to ask whether the participants were infected in the survey for ethical reasons. Instead, we asked whether they received any in‐hospital or home treatment for 1 week or longer in the past 6 months to exclude respondents who were potentially infected. As a sensitivity analysis, we conducted the same analysis excluding participants who reported hospital admissions or home treatment for 1 week or longer during the past 6 months. The primary outcome was the total number of adverse reactions.

Multiple logistic regression analysis was conducted to examine the relationship between four psychosocial factors at work and severe adverse reactions requiring medical care after getting vaccinated (i.e., anaphylaxis). SPSS 28.0 (IBM Corp.) Japanese version was used. Statistical significance was set as a two‐sided *P* < .05.

## 3 RESULTS

The participants' characteristics are shown in Table 1. The mean age was 44.8 years old (min‐max: 22–62 years old). Those with chronic disease accounted for 14.5%, and 8.6% of the participants experienced hospital admissions or home treatment over 1 week regardless of COVID‐19 during the past 6 months.

**TABLE 1:** Participants' sociodemographic characteristics and psychosocial factors at work at baseline (*N* = 747).

Variables [possible range]	*N* (%)	Mean (SD)
Age		44.8 (10.2)
20–29 years old	62 (8.3)	
30–39 years old	173 (23.2)	
40–49 years old	233 (31.2)	
50+	279 (37.3)	
Gender		
Men	423 (56.6)	
Women	324 (43.4)	
Marital status		
Single	320 (42.8)	
Married	427 (57.2)	
Educational attainment^a^		
Less than a high school diploma	145 (19.3)	
College/vocational	16.2 (16.2)	
Undergraduate	296 (39.6)	
Graduate+	37 (5.0)	
Unknown/others	148 (19.8)	
Occupation^b^		
Managers	93 (12.4)	
Non‐manual workers	447 (59.8)	
Manual workers	184 (24.6)	
Healthcare workers	23 (3.1)	
Chronic disease^c^		
None	639 (85.5)	
Any	108 (14.5)	
Hospital admissions/home treatment^d^		
No	683 (91.4)	
Yes	64 (8.6)	
The number of vaccinations		
First shot	60 (8.0)	
Second shot	687 (92.0)	
Type of vaccine		
Pfizer/BioNTech	484 (64.8)	
Moderna	246 (32.9)	
Unknown	17 (2.3)	
Anxiety about adverse reactions of vaccination [1–4]		3.0 (0.8)
Fear and worry about COVID‐19 [1–6]		4.3 (1.2)
Job demand [3–12]		7.9 (2.3)
Job control [3–12]		8.0 (2.2)
Coworker support [3–12]		7.0 (2.2)
Supervisor support [3–12]		6.7 (2.2)
Psychological distress [0–24]		5.5 (5.7)

Abbreviation: SD, standard deviation.

^a^ Information about educational attainment was obtained in May 2020.

^b^ Information about the occupation was created by merging the data obtained in February 2019 with information of the job category (i.e., healthcare workers or not) in May 2020.

^c^ Chronic disease refers to having any physical and psychological diseases currently treated in the medical settings, including hypertension, diabetes, heart disease (e.g., angina, heart failure), cerebrovascular disease (e.g., cerebral infarction, cerebral hemorrhage), cancer/malignant neoplasm, respiratory disease, liver disease, kidney disease, and depression/anxiety/unstable moods.

^d^ Hospital admissions or home treatment over 1 week, regardless of COVID‐19, in the past 6 months.

The prevalence rates of self‐reported adverse reactions after getting a COVID‐19 vaccine are shown in Table 2. The most prevalent adverse reactions were arm pain/redness/swelling (81.1%), fatigues/tiredness (64.1%), muscle pains/joint pains (63.3%), and fever (37.5°C +) (53.5%). In contrast, severe reactions requiring needed medical care (e.g., anaphylaxes) accounted for 2.9%+ (53.5%). In contrast, severe reactions requiring needed medical care (e.g., anaphylaxes) accounted for 2.9%.

**TABLE 2:** Prevalence of self‐reported adverse reactions after getting a COVID‐19 vaccine.

Variables [possible range]	Entire sample (*N* = 747)	
	*N* (%)	Mean (SD)
Total number of side reactions [0–11]		3.8 (2.2)
Arm pain/redness/swelling	606 (81.1)	
Fatigues/tiredness	479 (64.1)	
Headache	295 (39.5)	
Muscle pains/joint pains	473 (63.3)	
Chills	239 (32.0)	
Fever (37.5°C+)	400 (53.5)	
Nausea/vomit	50 (6.7)	
Diarrhea	55 (7.4)	
Lymph node pain	67 (9.0)	
Severe reactions to be needed medical care (e.g., anaphylaxes)	22 (2.9)	
Delayed local arm reactions after 7 days of vaccinations (i.e., COVID arm)	139 (18.6)	

Abbreviation: SD, standard deviation.

The results of multiple linear regression analysis are shown in Table 3. Coworker support was significantly and negatively associated with the number of adverse reactions in Model 2 and Model 3 (standardized *β* = −0.101, *P* = .021; *β* = −0.100, *P* = .023, respectively). Psychological distress at baseline was significantly associated with adverse reactions in Model 3 (*β* = .115, *P* = .002). Women, younger age, second vaccination, and Moderna were significantly associated with the number of the adverse reactions.

**TABLE 3:** Association between psychosocial factors at work and the total number of adverse reactions of COVID‐19 vaccines in the entire sample (*N* = 747): multiple linear regression analysis.

	Model 1				Model 2				Model 3			
	*B*	SE	*β* ^a^	*P*	*B*	SE	*β* ^a^	*P*	*B*	SE	*β* ^a^	*P*
Job demand (score, 3–12)	0.073	0.034	0.079	.032*	0.056	0.032	0.060	.080	0.024	0.032	0.026	.461
Job control (score, 3–12)	−0.062	0.039	−0.062	.114	−0.042	0.036	−0.042	.251	−0.017	0.036	−0.017	.643
Coworker support (score, 3–12)	−0.117	0.048	−0.115	.016*	−0.103	0.045	−0.101	.021*	−0.102	0.045	−0.100	.023*
Supervisor support (score, 3–12)	0.054	0.049	0.055	.264	0.020	0.045	0.020	.661	0.039	0.045	0.039	.386
Gender (women vs. men)					0.798	0.172	0.181	<.001*	0.725	0.174	0.164	<.001*
Age (years)					−0.046	0.008	−0.213	<.001*	−0.043	0.008	−0.199	<.001*
Education (university+ vs. less than university)^b^					0.078	0.159	0.018	.624	0.101	0.157	0.023	.521
Marital status (married vs. single)					0.187	0.163	0.042	.253	0.195	0.162	0.044	.228
Occupation (ref: managers)												
Non‐manual workers					0.070	0.253	0.016	.783	−0.010	0.251	−0.002	.969
Manual workers					0.033	0.281	0.006	.908	−0.098	0.280	−0.019	.725
Healthcare workers					0.284	0.475	0.022	.550	0.204	0.472	0.016	.665
Chronic disease (any vs. none)					0.272	0.214	0.044	.204	0.096	0.216	0.015	.657
Vaccination (second vs. first time)					1.398	0.275	0.174	<.001*	1.415	0.272	0.176	<.001*
Type of vaccine (ref: Pfizer/BioNTech)												
Moderna					1.227	0.160	0.264	<.001*	1.245	0.158	0.268	<.001*
Unknown					0.908	0.493	0.062	.066	0.941	0.490	0.064	.055
Anxiety about adverse reactions of vaccination (score, 1–4)									0.122	0.100	0.046	.223
Fear and worry about COVID‐19 (score, 1–6)									0.112	0.067	0.063	.093
Psychological distress (K6 score, 0–24)									0.044	0.014	0.115	.002*

*Note*: Eleven adverse reactions after vaccination were assessed: arm pain/redness/swelling, fatigues/tiredness, headache, muscle pains/joint pains, chills, fever (37.5°C+), nausea/vomit, diarrhea, lymph node pain, severe reactions requiring medical care (e.g., anaphylaxis), and delayed local arm reactions after 7 days of vaccinations (i.e., COVID arm).

Abbreviation: SE, standard error.

^a^ Standardized beta.

^b^ Educational attainment was dichotomized into two categories. Missing or unknown was classified as less than university attainment.

* *P* < .05.

The subgroup analysis with participants who received second vaccination during the follow‐up (*N* = 687) showed the negative associations of coworker support with the total number of adverse reactions in Model 3 (*β* = −0.094, *P* = .045) (Table A1).

The result of multiple linear regression analysis among those who did not experience hospital admissions or home treatment during the past 6 months (*N* = 683) is shown in Table A2. In Model 3, coworker support was significantly negatively associated with the number of adverse reactions (*β* = −0.109, *P* = .017).

The result of multiple logistic regression analysis with the presence of severe adverse reactions (e.g., anaphylaxis, one item) in the entire sample (*N* = 747) is shown in Table A3. In Model 3, supervisor support was significantly positively associated with the presence of severe adverse reaction (adjusted odds ratio = 1.36, *P* = .027). A similar analysis among those who did not experience hospital admissions or home treatment during the past 6 months (*N* = 683) is shown in Table A4. None of the four psychosocial working conditions correlated significantly with the presence of severe adverse reactions (one item) in the adjusted model.

## 4 DISCUSSION

High coworker support was negatively associated with the number of adverse reactions of the COVID‐19 vaccinations in this sample of employees, after adjusting for demographic variables, the number of vaccinations and type of vaccine, as well as their worries about COVID‐19 infection and adverse reactions by vaccination at baseline. Job demand, control, or supervisor support did not show significant associations with the number of adverse reactions. High psychological distress was associated with adverse reactions. This study demonstrated the importance of coworker support in experiencing adverse reactions after the COVID‐19 vaccinations among employees.

Coworker support showed a significant negative association with the total number of adverse reactions of the COVID‐19 vaccine. The finding is consistent with previous findings showing that psychological sfactors, i.e., exposure to a stressor, precipitate immediate inflammatory reactions to vaccines^4^, ^7^ and that poor psychosocial working conditions often increase inflammatory markers.^16^ However, this association was observed even after adjusting for baseline psychological distress, fear, and worry about COVID‐19. Although psychological distress may partially mediate the association, poor coworker support may be independently associated with the side effect of the COVID‐19 vaccine. The possible mechanism underlying the association is unclear, but the finding that the lack of social support generally increases systematic inflammations might clarify this mechanism.^4^, ^12^, ^28^, ^29^ Mediators and products that cause inflammation in the circulation can affect body systems to cause systemic adverse reactions.^30^ For instance, a previous study indicated that low coworker support influenced inflammation biomarkers in a group of employees who worked more than 41 h per week.^12^ It may be plausible that elevated inflammatory responses associated with lack of coworker support may exaggerate an innate immune response to the COVID‐19 vaccine.^31^, ^32^

Job demand, control, or supervisor support showed no significant associations with reported adverse reactions. These psychosocial working conditions are also related to chronic inflammations; for example, previous studies have indicated that high supervisor support was associated with low inflammation markers.^12^, ^33^ These could also potentially affect the adverse reactions. The statistical power to detect these associations may be limited due to the small sample size. However, the most puzzling observation was that supervisor support correlated with adverse reactions slightly positively. This may be due to a bias due to a selective attrition that participants who experienced lower supervisor support and a greater number of adverse reactions could be more likely lost to follow‐up. In addition, supervisor support was significantly positively associated with the severe adverse reaction, while this pattern was not significant among participants without hospital admissions/home treatment during the past 6 months. Thus, the finding may be partly due to confounding by a poor health status before the baseline which may require support from their supervisors and was associated with the development of a severe adverse reaction. A beneficial effect of supervisor support on preventing adverse reactions, if any, may have been masked by such the attrition or confounding of a pre‐existing health condition in this study. Future investigations should use larger samples to replicate the findings, with a more detailed measure of different (for instance, instrumental and emotional) aspects of supervisor support, controlling for pre‐existing health conditions.

### 4.1 Strengths and limitations

The strength of this study was the prospective nature of the study design. This study showed direct and indirect effects of the psychosocial working conditions on the immune function. However, the present study had several limitations. First, self‐reported measures were used to assess the psychosocial working conditions and adverse reactions. People with poor working conditions or high distress may have overreported the adverse reactions. Besides, employees with low coworker support may be easily conscious of adverse reactions because fewer people around them take over their work, so they are more concerned about the impact on their work. Second, this study did not consider other psychosocial conditions other than job demand, job control, and workplace social support. Other factors should be investigated in future research. Third, generalizability was limited because the participants were full‐time employees in Japan, and the data were retrieved from the online panel, although we selected samples to make that sex, and age were equally collected without limiting the prefectures of Japan. Compared to the Japanese representative public data (Labour Force Survey), the present cohort had a slightly higher proportions of managers and non‐manual workers. Less number of manual and healthcare workers also caused generalizability. The prevalence of each adverse reaction was similar to that reported in a previous Japanese study.^34^ However, it was still possible that participants under‐ or over‐reported some symptoms. Fourth, selection bias may occur due to the attrition during the follow‐up, while the follow‐up rate was relatively high. Fifth, the significant association found in this study could have been superficial, as other potential confounding factors could have affected the results. Sixth, this study addressed only a very short‐term innate immune response to vaccination but did not examine the effects on cellular or humoral immunity after vaccination and over longer period. The previous meta‐analysis revealed a negative association between stress and antibody production after influenza vaccinations.^35^ In addition, several studies have suggested that lonely or socially isolated individuals had a weak immune response after vaccinations^4^, ^36^, ^37^ and increased susceptibility to infectious disease.^38^ Future studies can examine the association of psychosocial working conditions and psychological stress with decreased responses to vaccinations and infectious susceptibility.

### 4.2 Implications for future practice

This study demonstrated the importance of psychosocial working conditions, especially coworker support, in experiencing certain adverse events after the COVID‐19 vaccinations. Informing employees about the potential effects of low coworker support on adverse vaccine outcomes may help them prepare for these adverse reactions. Improving coworker support in the workplace may reduce some of the reported adverse reactions. Active actions to control the reactogenicity may ease the vaccine hesitancy. Even before COVID‐19, stressors and stress reactions have been known to be important factors in immune functions. Accordingly, they should receive even greater attention during the pandemic. Further studies are needed to examine the association of social supports with immune responses.

## 5 CONCLUSIONS

Coworker support showed a significantly negative association with experiencing short‐term adverse reactions. Providing information about the findings may help employees cope and prepare for potential adverse reactions. Future research is needed to replicate the findings with a large sample size.

## AUTHOR CONTRIBUTIONS

Norito Kawakami oversaw this study, supervising the process, and providing his expert opinion. Natsu Sasaki and Norito Kawakami organized the study design and analyzed the data. Collaborators Reiko Kuroda, Kanami Tsuno, and Kotaro Imamura ensured that questions related to the accuracy or integrity of any part of the work were appropriately investigated and resolved. All authors participated in administering the survey. Natsu Sasaki and Norito Kawakami wrote the first draft of the manuscript, and all other authors critically revised it. All authors approved the final version of the manuscript.

## FUNDING INFORMATION

The 2021 Health, Labour and Welfare Policy Research Grants; The Japan Ministry of Health, Labour, and Welfare; and an internal fund of the Department of Mental Health, Graduate School of Medicine at the University of Tokyo, supported this work. The sponsors had no role in the design and procedures of the study; collection, management, analysis, and interpretation of the data; the preparation, review, or approval of the manuscript; and the decision to submit the manuscript for publication.

## CONFLICT OF INTEREST

NK received grants from SB AtWork Corp, Fujitsu Ltd, and TAK Ltd, personal fees from the Occupational Health Foundation, SB AtWork Corp, RIKEN, Japan Aerospace Exploration Agency (JAXA), Japan Dental Association, Sekisui Chemicals, Junpukai Health Care Center, Osaka Chamber of Commerce and Industry, outside the submitted work. RK received grants from Grant‐in‐Aid for Young Scientists (B) from Japan Society for the Promotion of Science (JSPS), personal fees from SATORI electric CO., LTD, NXP Semiconductors, RIKEN, Toyotsu Chemiplas, Mitsubishi Materials Corporation outside the submitted work.

## DISCLOSURE

Approval of the research protocol: The Research Ethics Committee of the Graduate School of Medicine/Faculty of Medicine, The University of Tokyo, approved this study, No. 10856‐(2)(3)(4)(5).

Informed consent: Online informed consent was obtained from all participants after full disclosure and explanation of the purpose and procedures of this study. We explained that their participation was voluntary, and they could withdraw consent for any reason simply by not completing the questionnaire.

Registry and registration number of the study/trial: N/A.

Animal studies: N/A.

## Data Availability

The data supporting this study's findings are available from the corresponding author, NK, upon reasonable request.

## Appendix

**TABLE A1:** Association between psychosocial factors at work and the total number of adverse reactions among a subgroup of participants who took the second COVID‐19 vaccination during the follow‐up (*N* = 687): multiple linear regression analysis.

	Model 1				Model 2				Model 3			
	*B*	SE	*β* ^a^	*P*	*B*	SE	*β* ^a^	*P*	*B*	SE	*β* ^a^	*P*
Job demand (score, 3–12)	0.071	0.036	0.076	.048	0.064	0.034	0.067	.060	0.029	0.035	0.031	.402
Job control (score, 3–12)	−0.075	0.041	−0.075	.069	−0.046	0.038	−0.046	.233	−0.021	0.038	−0.021	.588
Coworker support (score, 3–12)	−0.099	0.051	−0.096	.053	−0.098	0.048	−0.095	.040*	−0.096	0.048	−0.094	.045*
Supervisor support (score, 3–12)	0.048	0.051	0.048	.351	0.019	0.048	0.019	.690	0.043	0.048	0.043	.372
Gender (women vs. men)					0.834	0.182	0.189	<.001*	0.756	0.183	0.171	<.001*
Age (years)					−0.050	0.008	−0.232	<.001*	−0.047	0.008	−0.215	<.001*
Education (university+ vs. less than university)^b^					0.083	0.168	0.019	.622	0.110	0.166	0.025	.510
Marital status (married vs. single)					0.202	0.174	0.046	.245	0.205	0.172	0.046	.233
Occupation (ref: managers)												
Non‐manual workers					−0.008	0.265	−0.002	.976	−0.106	0.263	−0.023	.688
Manual workers					0.080	0.297	0.016	.787	−0.076	0.295	−0.015	.798
Healthcare workers					0.458	0.523	0.034	.382	0.371	0.519	0.028	.475
Chronic disease (any vs. none)					0.203	0.225	0.033	.367	0.001	0.227	0.000	.995
Type of vaccine (ref: Pfizer/BioNTech)												
Moderna					1.246	0.167	0.269	<.001*	1.266	0.165	0.274	<.001*
Unknown					0.885	0.501	0.063	.078	0.911	0.497	0.065	.068
Anxiety about adverse reactions of vaccination (score, 1–4)									0.156	0.106	0.058	.142
Fear and worry about COVID‐19 (score, 1–6)									0.096	0.070	0.054	.172
Psychological distress (K6 score, 0–24)									0.049	0.015	0.128	<.001*

*Note*: Eleven adverse reactions were reported after vaccination: arm pain/redness/swelling, fatigues/ tiredness, headache, muscle pains/joint pains, chills, fever (37.5°C+), nausea/vomit, diarrhea, lymph node pain, severe reactions requiring medical care (e.g., anaphylaxis), and delayed local arm reactions after 7 days of vaccinations (i.e., COVID arm).

Abbreviation: SE, standard error.

^a^ Standardized beta.

^b^ Educational attainment was dichotomized into two categories. Missing or unknown was classified as less than university attainment.

* *P* < .05.

**TABLE A2:** Association between psychosocial factors at work and the total number of adverse reactions after COVID‐19 vaccination among participants without hospital admissions/home treatment (*N* = 683): multiple linear regression analysis.

	Model 1				Model 2				Model 3			
	*B*	SE	*β* ^a^	*P*	*B*	SE	*β* ^a^	*P*	*B*	SE	*β* ^a^	*P*
Job demand (score, 3–12)	0.074	0.035	0.081	.034*	0.056	0.032	0.061	.083	0.020	0.033	0.021	.550
Job control (score, 3–12)	−0.076	0.041	−0.078	.060	−0.059	0.037	−0.06	.113	−0.027	0.037	−0.027	.471
Coworker support (score, 3–12)	−0.120	0.051	−0.118	.019*	−0.107	0.047	−0.105	.022*	−0.111	0.047	−0.109	.017*
Supervisor support (score, 3–12)	0.069	0.051	0.070	.177	0.042	0.047	0.042	.377	0.065	0.047	0.065	.164
Gender (women vs. men)					0.739	0.179	0.168	<.001*	0.654	0.180	0.149	<.001*
Age (years)					−0.048	0.008	−0.221	<.001*	−0.045	0.008	−0.208	<.001*
Education (university+ vs. less than university)^b^					0.039	0.165	0.009	.815	0.058	0.162	0.013	.720
Marital status (married vs. single)					0.113	0.170	0.026	.507	0.12	0.167	0.027	.473
Occupation (ref: managers)												
Non‐manual workers					−0.012	0.261	−0.003	.963	−0.087	0.258	−0.019	.737
Manual workers					−0.043	0.291	−0.009	.882	−0.182	0.287	−0.036	.526
Healthcare workers					0.628	0.536	0.045	.242	0.568	0.530	0.041	.284
Chronic disease (any vs. none)					0.207	0.224	0.033	.354	0.008	0.223	0.001	.972
Vaccination (second vs. first time)					1.360	0.304	0.159	<.001*	1.342	0.300	0.157	<.001*
Type of vaccine (ref: Pfizer/BioNTech)												
Moderna					1.305	0.167	0.28	<.001*	1.340	0.165	0.288	<.001*
Unknown					0.665	0.505	0.046	.188	0.718	0.499	0.050	.151
Anxiety about adverse reactions of vaccination (score, 1–4)									0.129	0.104	0.049	.219
Fear and worry about COVID‐19 (score, 1–6)									0.142	0.068	0.081	.038*
Psychological distress (K6 score, 0–24)									0.051	0.015	0.132	<.001*

*Note*: Eleven were observed after vaccination: arm pain/redness/swelling, fatigues/ tiredness, headache, muscle pains/joint pains, chills, fever (37.5°C+), nausea/vomit, diarrhea, lymph node pain, severe reactions requiring medical care (e.g., anaphylaxis), and delayed local arm reactions after 7 days of vaccinations (i.e., COVID arm).

Abbreviation: SE, standard error.

^a^ Standardized beta.

^b^ Educational attainment was dichotomized into two categories. Missing or unknown was classified as less than university attainment.

* *P* < .05.

**TABLE A3:** Association between psychosocial factors at work and severe adverse reaction (e.g., anaphylaxis) of COVID‐19 vaccines: multiple logistic regression analysis in the entire sample (*N* = 747).

	Model 1			Model 2			Model 3		
	OR	95% CI	*P*	OR	95% CI	*P*	OR	95% CI	*P*
Job demand (score, 3–12)	0.98	0.81–1.18	.814	0.96	0.80–1.17	.702	1.01	0.83–1.23	.918
Job control (score, 3–12)	0.97	0.78–1.19	.748	0.97	0.79–1.20	.768	0.95	0.76–1.18	.643
Coworker support (score, 3–12)	0.79	0.61–1.02	.074	0.83	0.63–1.08	.170	0.82	0.62–1.09	.170
Supervisor support (score, 3–12)	1.37	1.06–1.77	.015*	1.37	1.05–1.80	.020*	1.36	1.04–1.79	.027*
Gender (women vs. men)				1.77	0.64–4.89	.273	2.16	0.74–6.33	.161
Age (years)				0.99	0.94–1.04	.619	0.99	0.94–1.04	.575
Education (university+ vs. less than university)^a^				0.61	0.22–1.67	.336	0.60	0.22–1.66	.327
Marital status (married vs. single)				1.51	0.58–3.95	.402	1.65	0.62–4.40	.315
Occupation (ref: managers)									
Non‐manual workers				1.98	0.22–17.91	.544	2.37	0.26–21.80	.445
Manual workers				2.80	0.29–27.35	.377	3.50	0.35–35.23	.288
Healthcare workers				3.22	0.17–61.12	.436	3.47	0.18–67.27	.411
Chronic disease (any vs. none)				0.71	0.15–3.31	.663	0.97	0.20–4.67	.965
Vaccination (second vs. first time)				1.60	0.20–12.93	.657	1.53	0.19–12.42	.692
Type of vaccine (ref: Pfizer/BioNTech)									
Moderna				1.25	0.47–3.34	.651	1.23	0.46–3.31	.682
Unknown				7.48	1.75–31.91	.007*	7.08	1.60–31.32	.010*
Anxiety about adverse reactions of vaccination (score, 1–4)							0.72	0.40–1.29	.263
Fear and worry about COVID‐19 (score, 1–6)							0.89	0.61–1.30	.541
Psychological distress (K6 score, 0–24)							0.97	0.89–1.06	.464

*Note*: Severe adverse reaction was defined as self‐reported severe reactions requiring medical care after vaccination (e.g., anaphylaxis). Severe adverse effects were observed in *n* = 22 (of the entire sample) and *n* = 16 (of the participants without hospital admissions/home treatment).

Abbreviations: CI, confidence interval; OR, odds ratio.

^a^ Educational attainment was dichotomized into two categories. Missing or unknown was classified as less than university attainment.

* *P* < .05.

**TABLE A4:** Association between psychosocial factors at work and severe adverse reaction (e.g., anaphylaxis) after COVID‐19 vaccines: Multiple logistic regression analysis of participants without hospital admissions/home treatment (*N* = 683).

	Model 1			Model 2			Model 3		
	OR	95% CI	*P*	OR	95% CI	*P*	OR	95% CI	*P*
Job demand (score, 3–12)	0.94	0.76–1.17	0.590	0.94	0.75–1.17	0.569	0.99	0.78–1.25	0.925
Job control (score, 3–12)	0.93	0.73–1.18	0.544	0.96	0.75–1.22	0.718	0.94	0.73–1.22	0.649
Coworker support (score, 3–12)	0.82	0.61–1.11	0.198	0.88	0.65–1.19	0.411	0.88	0.64–1.20	0.412
Supervisor support (score, 3–12)	1.42	1.05–1.90	0.022*	1.36	1.01–1.85	0.045*	1.35	0.99–1.84	0.057
Gender (women vs. men)				1.74	0.53–5.73	0.366	2.17	0.60–7.88	0.237
Age (years)				0.94	0.89–1.00	0.056	0.94	0.89–1.00	0.046
Education (university+ vs less than university)^a^				0.69	0.21–2.22	0.531	0.69	0.21–2.25	0.533
Marital status (married vs. single)				1.05	0.34–3.25	0.929	1.17	0.37–3.72	0.791
Occupation (ref: managers)									
Non‐manual workers				0.72	0.07–7.75	0.787	0.87	0.08–9.42	0.905
Manual workers				1.37	0.12–15.70	0.798	1.64	0.14–19.31	0.695
Healthcare workers				2.81	0.14–54.92	0.496	2.73	0.14–54.98	0.512
Chronic disease (any vs. none)				0.55	0.07–4.63	0.586	0.81	0.09–7.09	0.845
Type of vaccine (ref: Pfizer/BioNTech)									
Moderna				0.95	0.27–3.28	0.931	0.86	0.24–3.03	0.813
Unknown				9.86	2.20–44.26	0.003*	8.86	1.87–41.91	0.006*
Anxiety about adverse reactions of vaccination (score, 1–4)							0.62	0.31–1.24	0.178
Fear and worry about COVID‐19 (score, 1–6)							1.01	0.65–1.56	0.978
Psychological distress (K6 score, 0–24)							0.97	0.87–1.08	0.517

*Note*: Severe adverse reaction was defined as self‐reported severe reactions requiring medical care after vaccination (e.g., anaphylaxis). Severe adverse effects were observed in *n* = 22 (of the entire sample) and *n* = 16 (of the participants without hospital admissions/home treatment). The number of vaccinations was excluded from the analysis due to an unstable result which was caused small sample of first time shot.

Abbreviations: CI, confidential interval; OR, odds ratio.

^a^ Educational attainment was dichotomized into two categories. Missing or unknown was classified as less than university attainment.

* *P* < .05.
